# Consensus Democracy: The Swiss System of Power-Sharing

**DOI:** 10.1007/978-3-030-63266-3_5

**Published:** 2021-01-21

**Authors:** Wolf Linder, Sean Mueller

**Affiliations:** 3 Department of Political Science, University of Bern, Bern, Switzerland; 4 Institute of Political Science, University of Lausanne, Lausanne, Switzerland

## Abstract

This chapter unpacks the notion of power-sharing and explains its centrality for political Switzerland. While direct democracy has played an important part in its evolution, law-making in Switzerland has become impossible without the participation of various interest groups at early stages of drafting already. The chapter details the functioning of Switzerland’s broad-based political pluralism, its structure of consociational democracy, the representation of the most important political parties and interest groups, and the ensuing processes of negotiation and mutual adjustment. It also discusses challenges and pitfalls of power-sharing.

## The Development of Swiss Consensus Democracy

In earlier chapters, we have already mentioned some elements of power-sharing, consociational or consensus democracy, which the Swiss call ‘system of concordance’. Its two main characteristics are the following: first, the executive is composed of a grand coalition. The goal is to let all important political forces participate in governmental politics, and to share the political responsibility with all these forces. Second, political behaviour within this grand coalition is geared towards permanent negotiation and compromise.

Power-sharing or consensus democracy is not unique to Switzerland. Variants of it can also be found in countries as different as Belgium, The Netherlands, India or South Africa. Power-sharing democracy is usually contrasted to the predominant, Anglo-American model of majoritarian democracy , in which the government is composed of a simple majority, holds all political power and can impose its decisions onto the minority. We return to this topic in Chap. 10.1007/978-3-030-63266-3_6. Here, we describe the Swiss power-sharing institutions, their development and functioning, and their strong points and weak spots.

If you ask the Swiss today why they like power-sharing, a typical answer is: ‘I find it fair that all languages, regions and political parties are represented in the government. This is better for our country because Switzerland needs political compromises rather than majority decisions’. History tells us, however, that in 1848 the Swiss Constitution was partly conceived as a majoritarian democracy . For several decades a single party, the Radicals, held all the power in a majoritarian regime. The development of power-sharing institutions and practice came only later. Three factors favoured the institutional conversion of the majoritarian regime into a power-sharing system.

The first is federalism. The small, rural, mostly Catholic cantons had a veto position in federal decision-making right from the beginning. In a coalition with the French-speaking cantons, they were able to block a centralising project of a fully revised Constitution in 1872. This forced the ruling Radicals to seek political compromises for the successfully revised Constitution in 1874 (Linder et al. 2010; Swissvotes 2019). The second is the switch to a proportional electoral system in 1918/1919, which was the success of an alliance of Catholic-Conservatives and Social-Democrats fighting Radical predominance. As a consequence, the latter lost their parliamentary majority and the party system became increasingly fragmented. The third and most important factor is direct democracy. We have already mentioned, in Chap. 10.1007/978-3-030-63266-3_4, that the referendum is a strong incentive, even a constraint, to cooperate in the form of an oversized coalition because the risk of defeat in a popular vote is too high otherwise. This indirect, institutional effect of the referendum is as important as the direct effect of popular votes.

### The Impact of the Referendum on the Composition of the Government

The reader is reminded of the period following the introduction of the optional referendum in 1874 (see Chap. 10.1007/978-3-030-63266-3_4), when the Catholic-Conservative minority used the device like a machine gun to shoot down important projects of the Radical majority (Aubert 1974, 43–4). The governing party could see no other possibility than to come to an arrangement with the opposition. To integrate the Catholic minority , in 1891 the Radicals offered them one seat in their hitherto one-party government. The Conservatives accepted and henceforth had a voice in the Federal Council. But this also meant sharing political responsibility for the solutions proposed by the collegiate council. So, behind this ‘amicable agreement’ (Steiner 1974) there was coercive pressure to cooperate. The Radicals saw their large majority in parliament becoming useless if referendum challenges by the Catholic minority were not curbed. On the other hand, the Catholic minority , who were unlikely ever to obtain a parliamentary majority, could win more through partial cooperation with federal government projects than through systematic opposition.

Motivations for a similar integration of other important political forces led to ever wider power-sharing in the Federal Council. First, the Catholic-Conservatives obtained a second seat in 1908. In 1928, the farmers and burghers, who ten years before had split off from the liberal Radicals, were (re-)integrated through their own seat in the government. In 1935, the Social-Democrats became the largest political force in the National Council (27% of seats). Some cities even had left-wing majorities. But Social-Democratic claims for inclusion in the federal government were turned down by the bourgeois parties because of the prevailing class struggle. Only in 1943, when during World War II political integration and unity were needed more than ever, were the Socialists given their first seat. In 1959, following a short period with no Social-Democrat participation, the ‘ magic formula’ was born: until 2003, the Federal Council comprised two Radicals, two Christian-Democrats (formerly Catholic-Conservatives), two Social-Democrats and one member of the Swiss People’s Party (*Schweizerische Volkspartei* [SVP], formerly the Farmers’ and Burghers’ Party). After the 2003 elections, when the SVP became the largest party in the National Council (28% of seats) at the expense of other bourgeois parties, it received a second seat—at the cost of the Christian-Democrats, which corresponded to the logic of ‘arithmetic’ power-sharing in the government (e.g. Altermatt 2009; Vatter 2018, 218ff.). Except for 2008–2015 (see below, Sect. 5.5.1), this adjusted magic formula has remained intact (Table 5.1).

**Table 5.1:** Results of the government elections of 11 December 2019

Name	Party	Language	Canton	Gender	First elected in	Confirmed with^b^
Ueli Maurer	SVP	German	Zurich	Male	2008	213 votes
Simonetta Sommaruga	SPS	German	Bern	Female	2010	192 votes
Alain Berset	SPS	French	Fribourg	Male	2011	214 votes
Guy Parmelin	SVP	French	Vaud	Male	2015	191 votes
Ignazio Cassis	FDP	Italian	Ticino	Male	2017	145 votes
Viola Amherd	CVP	German	Valais	Female	2018	218 votes
Karin Keller-Sutter	FDP	German	St. Gall	Female	2018	169 votes
Walter Thurnherr^a^	CVP	German	Aargau	Male	2015	219 votes

^a^Federal Chancellor (=secretary general of the Federal Council)

^b^Total votes possible: 246

### Impacts on the Legislative Process

Integrating the main political parties into a governmental coalition was important; co-optation gave the newly represented parties in government a feeling of being recognised as equal. Co-optation, however, was not a free lunch but a deal: the parties of the more inclusive government coalition were expected to cooperate in parliament, supporting legislative compromises strong enough to survive a referendum. This was not always the case, and the lack of appropriate procedures for parliamentary compromise even led to a crisis of the Swiss political system.

In the period of worldwide economic depression in the 1930s, the bourgeois coalition not only came under pressure from the political left, but also from their ‘own’ interest groups who challenged bills put forth in parliament. Moreover, extremist forces, impressed by Nazi and fascist propaganda in Germany and Italy, tried to undermine trust in democracy and parliamentary institutions. Their so-called ‘ Frontist Initiative’, which proposed a radically new political order, was overwhelmingly rejected in a popular vote, but legislation became blocked by referenda challenges from all sides.

The Swiss political authorities had to learn that the referendum could also be successfully used by relatively small groups, and that it was difficult to obtain a sufficient majority even *with* the support of interest groups and parties. In the years before World War II, the Federal Assembly began to rely on the ‘urgency clause’ of Article 89 of the Constitution (now Art. 165), which authorises parliament to pass laws without a referendum when rapid decisions are required. Bypassing the ordinary legislative procedure in this way helped Switzerland to overcome the economic crisis of the 1930s. Democratic movements, however, criticised the utilisation of this clause, and a popular initiative in 1949 successfully restricted its scope (Box 5.1).

#### Box 5.1 Direct Democracy in Situations of Urgency and War^1^

Decision-making under direct democracy takes time, and its results can remain uncertain. How can the Swiss government cope with these difficulties in times of economic, security or public health crises, when rapid decision-making is necessary? We have to distinguish between two different mechanisms:

First, there is an ‘urgency (or emergency) clause’ in the Federal Constitution (Art. 165). It authorises parliament, if immediate action is necessary, to adopt laws without a referendum *(dringlich erklärte Bundesgesetze)*. After excessive use in the 1930s, the urgency clause was revised twice. A first amendment, introduced in 1939, restricted the terms of ‘urgency’ and required an absolute majority of members in each chamber. In 1949, a second amendment stipulated time limits. Under these rules, laws enter into force immediately but are limited in time. If a law has a constitutional base, it becomes subject to an optional and ‘abrogative referendum’. This means that the law is repealed after one year unless it is approved by the people. The Federal Assembly can even adopt urgent laws that are not based on the Constitution. Such an urgent federal act ‘must be repealed one year after being passed by the Federal Assembly if it has not in the meantime been approved by the People and the Cantons’ (Art. 165.3 FC 1999). Since 1949, therefore, direct democracy is no longer bypassed by the urgency clause , merely suspended for maximum one year.

In practice, the Federal Assembly still has a large interpretation of ‘urgency’. The old critique of Auer (1976) that the actual regulations still provide too much power to the Federal Assembly is thus justified. On the other hand, it cannot be denied that legislation under direct democracy needs too much time if parliamentary decisions have to be made in urgent situations such as natural catastrophes or—more recently—economic and public health disasters.

In addition to the urgency clause used by the Federal Assembly , the Swiss parliament delegated or recognised an urgency power of the Federal Council in times of World Wars I and II. These ‘full powers’—not mentioned in the Constitution of 1874 but given to the Federal Council in 1914 and 1939—comprised all measures necessary to ensure the survival of the population, notably regarding food supply. Using its ‘full powers’ during World War II, the Federal Council issued some 1800 emergency ordinances, whereas the Federal Assembly , in the same period from 1939 to 1945, adopted merely 220 laws and ordinances. The emergency ordinances of the Federal Council were subject to some control by parliament, but not to referenda. Today, Article 185 of the Constitution explicitly allows the Federal Council to ‘issue ordinances and rulings in order to counter existing or imminent threats of serious disruption to public order or internal or external security’. During the coronavirus pandemic, for instance, the Federal Council first relied on a special provision in the Epidemics Act but then also on Art. 185.

The authorities also developed mechanisms for a better integration of political parties, interest groups and the cantons into the law-making process. This *pre-parliamentary process* consists of two major stages. First, the Federal Council, when confronted with the need for new legislation, nominates a study group or committee of experts to evaluate the necessity and various options for new rules. The composition of these committees is worth mentioning. Some members may simply be experts, but most of them combine knowledge and power. The Federal Council strives to appoint members who represent the standpoints of the different groups eventually affected by the proposed legislation. Individual members may well have a reputation as experts on an issue, but the composition of the committee is made as representative as possible in order to cover all positions that could prove divisive during later discussions. On the basis of these expert deliberations, the ministry or office in charge of a project writes a first draft of the bill.

Second, there is a ‘consultation procedure’ (*Vernehmlassung*), open in principle to anyone. In practice, the federal administration circulates the first draft to all cantonal governments, political parties, the most important economic associations and other affected interest groups. However, it is the authorities who decide who is affected by a particular project, and the right to be consulted does not mean that the authorities accept the views put forth. Only after evaluating the responses from this procedure does the Federal Council decide whether to continue with the project. If the decision is made to go ahead, it is next sent to parliament.

Both elements of the pre-parliamentary process pursue the same goal: to reduce the risk of a referendum challenge or, in the case of the obligatory constitutional referendum, to reduce the chances of failure in the ensuing popular vote (Neidhart 1970; Papadopoulos 1999, 69–96; Blaser 2003).

## The System of Power-Sharing: Actors and the Political Process

### Actors and Their Functions

**Fig. 5.1:**
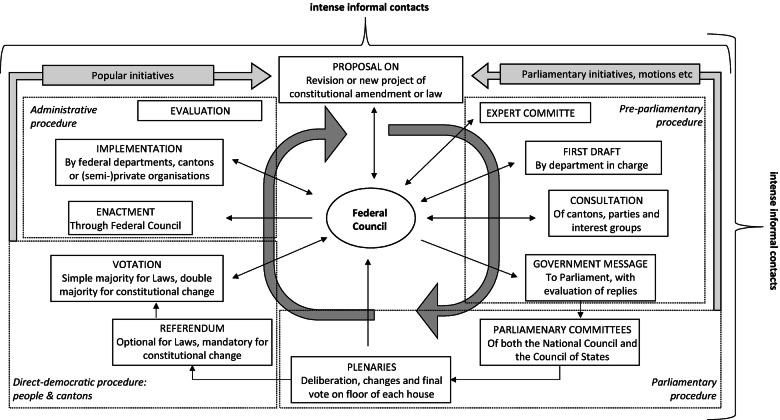
The legislative process: actors and the policy cycle

While in parliamentary democracies decision-making is concentrated in the parliamentary majority and the executive, the Swiss system of power-sharing is somewhat more complex: more actors are engaged who all, albeit with different functions, possess considerable influence. These actors have to cooperate, and we cannot find one sole centre of power. Figure 5.1 shows the main actors and illustrates the legislation process as a ‘policy cycle’. Let us start with the actors.

*Parliament:* According to the ideas of the fathers of the Constitution of 1848, the two chambers of parliament were the ‘highest authority’ of the federation. Indeed, until today parliament has a lot of power. Besides its main function of law-making, it elects the members of the Federal Council and the Federal Supreme Court, supervises the administration and can intervene in many ways. As there is no vote of confidence to bring down the government, parliament is free to criticise the projects of the Federal Council or even to reject them. Even so, the parliament has lost its institutional ‘supremacy’. Its freedom of action is restricted by direct democracy, by the interest groups who intervene in the pre-parliamentary process and by the Federal Council who largely controls the agenda in foreign policy and prepares most draft bills. Although on paper, the Swiss parliament is one of the most powerful comparatively, in practice it has only few resources to compete with the government and its administration, on the one hand, and business interests and civil society groups, on the other (Vatter 2018, 312ff.).

*Direct democracy:* Direct democracy began to play an important role when the people’s rights, originally restricted to the constitutional referendum, were extended to the optional referendum (1874) and then the popular initiative (1891). In Chap. 10.1007/978-3-030-63266-3_4 we discussed their influence at large.

*Interest groups:* Their prime arena of influence is the pre-parliamentary procedure, which was institutionally formalised after World War II. Note that participation in expert committees and the pre-parliamentary consultation is open not only to economic associations such as employer’s and trade unions, but also to other organisations, the cantons and even private individuals. We have already shown why in Switzerland interest groups have more influence in the pre-parliamentary phase than elsewhere: their additional bargaining power lies in the fact that they can use the referendum threat as a pawn. Moreover, interest groups often play an important role in implementation: the ‘ social partnership’ between labour and capital, or public-private partnerships once determined the design and execution of economic and social policies and remain important (see Box 5.2).

*Cantons:* In Switzerland, the 26 cantons are not only largely autonomous, more or less self-contained polities. They also, and increasingly, try to influence the federal government to act or refrain from acting in a certain way (e.g. Cappelletti et al. 2014; Schnabel and Mueller 2017). In doing so they profit from their strong position as part of the federal system, but also from the popular legitimacy of their governments and parliaments. The self-organisation of the cantonal governments in the form of a ‘Conference of Cantonal Governments’ has become an influential actor in Swiss politics. Cantonal representatives are regularly asked to sit on expert committees or attend parliamentary hearings. They can also directly petition the federal parliament using the cantonal initiative (e.g. Mueller and Mazzoleni 2016).

*The federal administration:* With the growth of the social and economic activities of the federation after World War II, the federal administration has acquired greater political influence, for two reasons. First, it has its own experts, who often direct the pre-parliamentary process. Second, it has all the feedback knowledge of implementation and evaluation, which in turn stimulates proposals for further legislative reform. In this way, the bureaucracy can also define its own interests.

*The Federal Council:* The main function of the Federal Council is the steering of the entire political process. Giving the go-ahead for most formal steps of decision-making, setting priorities in substance and time, the Federal Council has a great influence on the political agenda. It disposes of all the professional resources of the administration, which allow it to prepare its own policy projects. Political leadership of the Federal Council is limited, however, for two main reasons: consensus in an all-party government is difficult to achieve and often minimal in scope, while parliament, not obliged to support the government because there is no vote of confidence, can always turn down the propositions of the Federal Council. In foreign policy, however, the leadership of the Federal Council is more pronounced.

### The Policy Cycle

The policy cycle in Fig. 5.1 shows all the phases of the legislation process. It is conceived as an ongoing process of political problem-solving which starts with the first proposals for a new piece of legislation and provisionally ends with its implementation and evaluation. At every stage of the process, negotiations and the appropriate decisions may result in modifications, radical changes or even the abandonment of the project. If the programme enters its final phase, this is not the end: sooner or later the experiences with implementation will lead to new propositions for a reform, and the policy cycle begins anew.

*The pre-parliamentary procedure:* The cycle starts with propositions for a new law or a constitutional amendment. This can happen through a popular initiative, parliamentary instruments or by the administration, which is the informal gateway for pressure groups seeking reform. If the Federal Council initiates the process, it also organises the whole pre-parliamentary stage of the process. Depending on the issue, it charges the administration or mandates an expert committee to draft a first project. As most committee experts are also representatives of interest groups, this gives them a first chance to announce their position and voice opposition. The actors declare under which conditions they would support or fight the bill. This leads to mutual adjustments, for instance, between employers and trade unions on a social-security reform. The administration will defend its own views and interests but will also exercise a mediating role in conflicts not directly negotiable between antagonistic interests.

The subsequent consultation process involves further organisations, each formulating a position that represents the view of their members. When evaluating the results of this procedure, the administration seeks to maintain only those aspects of the reform that have found sufficient support and to avoid leaving actors worse off than before. If the (modified) draft fulfils these conditions, the Federal Council has good reasons to believe that the participating actors will support a constitutional amendment in the obligatory vote or refrain from an optional referendum in the much more frequent case of an ordinary legislative proposal. In the form of a ‘Message of the Federal Council’, the draft then enters the parliamentary procedure.

*The parliamentary procedure:* Each project has to find a majority in both chambers. If proceedings in the Council of State and the National Council end up with a difference in substance, negotiation procedures between the chambers are organised to align on the same solution. If this is not possible, the project has failed. For a long time in the twentieth century, the federal chambers had the reputation of being a weak parliament, accepting all too often and easily the compromises found between the vested interests in the pre-parliamentary phase. Today, such an appreciation is certainly wrong (cf. Sciarini et al. 2015, 34ff.). In the 1990s, parliament has greatly reformed its own organisation and procedures which led, among others, to a strengthening of its legislative committees. These now standing committees have become the centres of intense deliberation and negotiation. Empirical studies show that today, parliament modifies the projects of the Federal Council much more than it did before (Lüthi 1997; Jegher 1999; Vatter 2018, 302). Parliament also realises its own projects by means of a parliamentary initiative, which bypass some aspects of the pre-parliamentary process.^2^

As mentioned earlier (Chap. 10.1007/978-3-030-63266-3_4), less than 7% of law projects passed by parliament are challenged by an optional referendum. This means that the chambers seem to have a good flair for avoiding the referendum risk. This is due to several factors. The draft coming from the pre-parliamentary procedures has a story to tell: parliament knows which issues were controversial and which were accepted unanimously, and they are familiar with the positions of all-important actors, including the Federal Council. Many members of parliament have intense relations to interest groups whose points they support. The modifications of all phases of the procedure are documented for every article of the new bill. Thus, the members of parliament and its political groups know all about the difficulties and fragilities of any compromise that has been reached and can thus assess the robustness of a solution. The different parliamentary groups equally try to lower the risk of a referendum being called and look for a compromise that is supported by as many parties as possible.

*The direct-democratic procedure:* Our description of referenda campaign in Chap. 10.1007/978-3-030-63266-3_4 left one question open (Sciarini and Trechsel 1996): how come that a seemingly well-balanced project gives rise to opposition, and that opponents take the chance of an optional referendum? There are several answers to this question.

First, the referendum may be called by a governmental party. As already mentioned, consensus amongst the four of them is not always reached (Traber 2015). One or in rare cases two parties articulate their opposition in parliament, and a narrow majority, not giving in to the claims of the opposition, takes the risk or even accepts the challenge that its bill is tested in a popular vote. Sometimes the referendum is triggered by a cantonal branch of a political party despite the support of the governmental project by its national counterpart. This is because political parties are as ‘federal’ as the political system, and deviant positions of cantonal parties cannot be impeded by the national party. In similar situations and ways, the referendum is called by strong interest groups.

Second, also small political parties or even grass-roots movements are able to launch a referendum, and in rare cases they may even be successful. In 1969, for example, the students of the Swiss Federal Institute of Technology called a referendum against a new law on their university—and won (APS 1969). Even though the success of a small party or an outsider group is rare, they sometimes mobilise a considerable part of the electorate. This means that the compromise among political elites is not always accepted by ordinary people.

Third, if the consensus among political elites is fragile, a small outsider can initiate a chain reaction in which other actors or even governmental parties defect and join the referendum. This resembles a cargo ship loaded with barrels barely fastened. If one of the barrels gets loose and rolls from one board side to the other, many barrels will follow, and the ship keels over. These cases are hard to predict. Even though referendum cases make for less than 7% (one in about 15) of all legislative projects, they sometimes come as a surprise to the political elites.

The verdict of the people is binding and has immediate effect. In cases of referenda, the project is enacted or has failed. In cases of an accepted amendment to the Constitution, implementation may take more time if it needs an executing federal law , which has to pass a new policy cycle. This will be especially cumbersome if the amendment is due to a popular initiative accepted against a (large) majority among the political elites.

*The administrative procedure:* Once a project has received parliament’s assent and gotten the required majority in a popular vote, it enters into force. The implementation is an important part of the policy cycle (e.g. Varone 2007; Kissling-Näf and Wälti 2007; Sager et al. 2018). In many cases, policy programmes for proper implementation have to be developed or revised. As most programmes are implemented in close cooperation with the cantons, negotiations with their administrations take place. It is one of the characteristics of Swiss federalism that the national authorities have little means of coercion and greatly respect the autonomy and preferences of cantonal authorities in the implementation process. Resistance from the cantons may impede implementation. Conversely, negotiation and compromises may lead to intense cooperation, which in turn facilitates the implementation of federal policies. Thus, we may speak of a form of vertical power-sharing also at that stage. Evaluations, finally, may kickstart new policy cycles (cf. Sager et al. 2017).

#### Box 5.2 Social Partnership and Public-Private Partnerships—The Second Arenas of Power-Sharing

A) *Social partnership*

In the first decades of the twentieth century, industrial relations between labour and capital were characterised by class struggle , strikes and lockouts. This ended on the eve of World War II, when the Federal Council urged leaders of employers’ and labour organisations in the mechanical-engineering industry to resolve their conflicts by ways of negotiation and cooperation, which would better help to overcome the economic crisis. This marked the beginning of a new era: the ‘Labour Peace Convention’ (*Arbeitsfrieden*) of 1937 invited employers and unions to resolve all their conflicts through negotiation and to renounce on strike and lockouts. In the following decades, similar conventions were concluded in most other industries. Thus, industrial relations are characterised as ‘social partnership’ (*Sozialpartnerschaft*), leading to a typical pattern of social policy. Social policy was developed contractually between employers and unions, the circle of beneficiaries was restricted to the workforce in the respective industries. Both sides relied on the principle of ‘ subsidiarity’: state intervention should be the exception, restricted to those problems which social partnership was unable to resolve. This pattern of a ‘liberal welfare state’ has gradually changed in the last decades. As elsewhere, globalisation somewhat reduced the bargaining power of trade unions. As the contractual way became unfavourable for them, they changed their strategy and began to rely on legislation instead. The passage from contract to public law transformed Switzerland into a ‘normal’ welfare state, more and more responsible for all sorts of social policy. The unions were successful because their strategy of legislation let all people, not only those working in the industries, benefit from welfare and defend its benefits also by popular votations (Trampusch 2010). So far, employers and unions have kept social partnership in their own hands while the EU wants to gain control on Swiss industrial relations. This is presently one of the major issues of discussion in the negotiations between Switzerland and the EU, who both seek to put their cooperation on firmer grounds.

B) *Public-private partnerships*

Intensive cooperation between government and private actors is known in most economically developed democracies. It has a long tradition in Switzerland. As early as the late nineteenth century, private organisations, especially in agriculture, fulfilled certain functions for the federal government, which at the time lacked its own professional administration (Vatter 2018, 174). Indeed, Swiss governments at all levels sought to avoid building up a large bureaucracy. Whenever possible, the authorities preferred to use private organisations or create semi-private (parastatal) organisations to implement public policies. In agriculture, dozens of parastatal organisations proposed and policed regulations and organised the pricing, distribution and marketing of products (Jörin and Rieder 1985). The intensive cooperation between private organisations and the state is also known as ‘ neo-corporatism’. We doubt, however, whether one can speak of neo-corporatism in the case of Switzerland (see also Vatter 2018, 203ff.). Elsewhere, the term denotes tripartite arrangements between labour, capital and the state to regulate economic conflicts by concerted action. The Swiss case differs in many respects. First, there is no equilibrium of power between employers and unions: the latter are weaker. Second, concerted action is often avoided because arrangements are decentralised and vary considerably from sector to sector. Third, arrangements between private actors and the state also comprise social, cultural or environmental policies and are not always tripartite, but sometimes bilateral and competitive as in pluralism. It is thus more appropriate to speak of public-private partnerships.

Since the 1990s, public-private partnerships have changed considerably. With liberalisation and globalisation, many semi-public or parastatal organisations have disappeared or been privatised. This is particularly the case with agriculture, a domain where World Trade Organization (WTO) regulations forced Switzerland to abandon great parts of its traditional protectionism. While many public-private partnerships still exist or expand into new domains, liberalisation generally leads to a more restricted role of the ‘public’ and to the exposure of the ‘private’ to competition from both within and outside the country (Mach 2007, 2014).

## The Features of Power-Sharing

### The Main Characteristics of Political Compromise: No Single Winner Takes All, Everybody Wins Something

The entire political process aims at reaching a political compromise. Instead of a (small) majority that imposes its solution onto a (large) minority, we find mutual adjustment: no single winner takes all, everybody wins something. Some people attribute this behaviour to a specifically ‘Swiss’ culture. Indeed, there are some studies that show such differences: German economic elites, for instance, seek less compromise in conflict and use hierarchical power more than their Swiss counterparts (Kopper 1993). From a political science perspective, however, the effect of institutions seems to be paramount. The referendum challenge, the strong influence of cantons and interest groups as well as the multiparty system amount to formidable veto points that simply do not allow for majority decisions and compel political actors to cooperation and compromise. This means that every actor must renounce on some of their expectations, which is not always easy (Box 5.3).

#### Box 5.3 ‘No single winner takes all, everybody wins something’: Conditions of Good or Poor Compromise

*Context*

The idea that ‘no single winner takes all, everybody wins something’ has not always worked out. Mutual adjustments were most successful in the period leading up to the 1970s, when economic growth allowed the distribution of more public goods. In the aftermath of World War II—an experience that unified the small country—many old antagonisms between ideologies had disappeared. Optional referenda were few and the success rate of obligatory ones high.

Consensus became more difficult to achieve after the recession of the 1970s. With lower economic growth after the first oil crisis, there was less surplus to distribute. Political redistribution in social security and the health system became a zero-sum game: what one actor won another lost. Ecological sustainability became a political issue and prompted new conflicts and actors. The party system fragmented and new social movements arose. In conflicts over industrial and post-industrial values, and with the rise of neo-liberalism and neo-conservatism, part of the basic Swiss consensus melted away. At the end of the 1980s, important legislation failed or remained incomplete. In the last three decades, globalisation functioned as pressure from the outside, leading to quicker and larger steps of political innovation, but also to higher polarisation, the demarcation of winners and losers of internationalisation, and to the deepening of old cleavages such as that between cities and countryside.

*Issues*

The feasibility of ‘no single winner takes all, everybody wins something’, then, greatly depends on the specific issue. As long as money is involved, and as long as there is plenty available, compromises easily can be reached. But conflicts can also involve ‘indivisible’ goods. For example, in 1977 the Federal Council proposed to introduce daylight saving time in line with many West-European countries. Farmers refused to put their clocks one hour forward in spring and then back again in autumn, claiming their cows would produce less milk. The typical Swiss compromise was not feasible here: advancing the clock half an hour would have helped nobody. It was easy for the well-organised farmers to call for a referendum, and their challenge was successful. However, living on a ‘time isle’ in the centre of Europe was not very practical. Two years later, parliament passed a new bill and the farmers gave in. Similarly, compromise can be very difficult if an issue involves moral values such as abortion or same-sex marriage. Such topics are considered by many people to be a question of principle. Contrary to daylight saving time, pragmatic experience would not change preferences because interpretations will go both ways. In Switzerland, the abortion issue has led to several popular votations triggered by both sides. Neither proponents of liberalisation nor conservative opponents finding the status quo too liberal, however, could win a majority. Even the federalist idea of letting the cantons decide was rejected: while liberals accepted that cantons could practice different solutions, conservatives insisted that in no canton any liberalisation should be permitted. In other countries, such deadlocks are often solved when elections bring new majorities and a new government. Not so in Switzerland, where the government coalition rests the same. The deadlock lasted for more than 20 years before a solution was found. The example shows that mutual adjustment has its limits in Swiss politics, too.

*What is a good compromise?*

Obviously, there are compromises and compromises . A key distinction between good and bad compromises is not so much how many persons or groups can live with it, but rather who pays the price. Good compromises in this sense are those whose costs are shouldered by the actors participating in its forging. If employees agree to work more for the same pay but receive an extra week of holidays in exchange, both the costs and the benefits of the compromise accrue to them. If, however, their extra holiday is funded entirely by lower social benefits provided for future employees, the costs of the compromise are shifted onto groups without any representation and influence. That is one of the reasons why the Swiss consultation procedure foresees feedback from all affected sides, even if that is often difficult to achieve. It also means that to be heard, interests must organise politically.

### The Technique of Political Compromise: Compensations That Transform Conflict from Zero-Sum into Positive-Sum Games

Consensus, theoretically, requires a Pareto optimal solution in which no actor is left with losses. As illustrated in Box 5.3, it can be difficult or even impossible to meet this condition. In times of general budget cuts, for instance, compromises must distribute losses, and one of the few possibilities to reach consensus is a ‘ symmetry of sacrifices’, whereby each actor thinks that others agree to bear similar losses as she is willing to pay. But even under normal conditions, Pareto optimal solutions are not always at hand. Similar to residents around airports or along busy roads who are sometimes remunerated for their sacrifices in favour of others, actors in the political process receive compensation. Compensation is facilitated if the agenda of the issues to be negotiated is widened or the number of participants increased. The compromise reached under these circumstances may suffer from little effectiveness, though, if it violates the famous Tinbergen Rule: the Dutch economist Jan Tinbergen stated that ‘to successfully achieve *n* independent policy targets, at least the same number of independent policy instruments are required’ (Schaeffer 2019).

### Cooperation, Trust and the Deliberative Learning Processes

 Game theory shows that in a single game, self-interested actors defect from cooperation when it offers them an extra profit. This risk is considerably reduced if the same actors play many games. In this case, players may mutually sanction defection, which then becomes less attractive. This is exactly the case in a steady power-sharing arrangement, as it allows actors to develop mutual trust. An additional advantage for cooperation is found if politics involves different cleavages for which the opposed camps, for instance the left or the young, are not the same. This leads to different coalitions from issue to issue, cementing an important aspect of the ongoing process of power-sharing: political actors opposed today on a particular issue may find themselves as coalition partners tomorrow on a different issue. Mutual respect and amicable relations even with the opponent (of today) are the result.

Indeed, studies on deliberation provide empirical evidence that under conditions of power-sharing, political opponents have more respect for each other and listen more to the arguments of the other side than in majoritarian settings (Bächtiger et al. 2005; see also Bernauer and Vatter 2019). Thus, power-sharing allows for deliberative learning processes. The weak spot is, however, that conditions for changing coalitions are not always given. In the 1980s, for instance, the three parties of the centre-right regularly overruled the smaller green-left parties on major issues of public finance, energy and the environment. Behind the screen of all-party power-sharing, informal majoritarian politics were practised. This is a bad constellation—instead of combining advantages, informal majoritarian and formal power-sharing politics combine the disadvantages: the ruling majority refuses to compromise and is not exposed to the risk of losing power through competitive elections as would be the case in a majoritarian system. In such a position, the ‘eternal’ majority can afford not to learn—for Deutsch (1967) a pathological use of power.

### Political Elitism and Its Limits

Power-sharing produces strong formal and informal contacts amongst the entire political elite. Lijphart’s (1969) early theory of ‘ consociationalism’ proposes that power-sharing also leads to the development of common values and attitudes.^3^ Elites develop a common way of understanding problems which must be solved, and they learn to adopt perspectives that go beyond their specific group interests. Does this mean that power-sharing leads to a cartel of ‘the establishment’, neutralising electoral competition and abolishing democratic accountability? In the Swiss case, it may be argued that indeed elections do not lead to a change of roles between government and opposition and therefore play a minor role for democratic control. Direct democracy, however, is a strong corrective to elitist consociationalism. Every political party and its leaders have to regularly defend their decisions before a people’s vote. This imposes both procedural and substantive limits to elitism.

## The Critics of Swiss Consensus Democracy

### The Referendum as an Instrument of Vested Interests

We already showed that the referendum is a pawn in the hands of interest groups, giving them additional influence in all matters of legislation. Thus direct democracy, instead of being the voice of the people, has partly become the instrument of vested interests. Indeed, this critique has some traction, especially for the long period of time during which the Swiss parliament was weak and often adopted the pre-parliamentary compromise struck by interest groups without much modification. A famous constitutionalist went as far as to say that the law is no longer the result of parliamentary proceedings but of negotiating non-democratic, vested interests (Huber 1971).

Today, the image of a *Verbandsstaat*, that is, a state of vested interests that dominates parliament, corresponds much less to political reality. Not only has parliament become more vocal in shaping legislation (Sciarini et al. 2015; Sciarini 2007), but due to globalisation some of the strongest interest groups in the domestic market, namely those of agriculture and industry, have lost some influence. Also, many traditional coalitions, such as those in industry or between employers and unions, are split into trade-oriented versus protectionist, often neutralising each other.

### Inequalities of Influence

The weak spot of democratic pluralism is that it cannot guarantee fair competition in the sense that all interest groups and parties have the same chances of political influence. According to the theory of collective action (Olson 1965), the negotiating power of a group depends on two factors: its organisational ability (e.g. to mobilise members) and its capacity to deny contributions that other actors need. This leads to stark inequalities of influence. In negotiations and law-making by mutual adjustment, the ‘haves’ are better off than the ‘have-nots’, whose refusal to compromise remain without effect. Moreover, organisations which defend specific short-term benefits for their members are likely to be stronger than those promoting general and long-term interests.

Big companies, for instance, can easily mobilise against new regulations on their market. Their threat to leave Switzerland is a strong argument for parliament not to pass such a bill. Consumer organisations, on the other hand, have more difficulties. They constitute much larger but probably less powerful groups. Their interests may conflict, as can be shown in the case of genetic engineering: part of the consumers may favour genetically modified products, so only the other part will mobilise against them. The consumer organisations’ only means is the consumer boycott, rarely efficient in the short run. Therefore, they do not wield a plausible threat and possess no trade-in in negotiations. Environmental groups in particular face the problem of having to fight for a long-term public good from which nobody can be excluded, such as clean air and rivers. They are popular and outnumber the biggest political parties in membership. Faced with vested business interests, however, they are not able to articulate comparable threats.

On the whole, negotiations do not necessarily eliminate the twofold objections to political pluralism: the ‘haves’ retain their advantage over the ‘have-nots’, and negotiations amongst interest groups favour particular short-term benefits at the expense of general long-term interests (Scharpf 1970; Huber 1971, 589–630). One may object that these inequalities are not peculiar to Swiss semi-direct democracy but apply to all pluralist systems. What is more, negotiating in the shadow of direct democracy offers even weaker actors defending diffuse interests a chance to use the referendum as a last resort.

### Lack of Innovation?

Negotiation and compromise seem to have provided important advantages. In the absence of electoral change, there are no abrupt discontinuities in federal policy. The sobering effect of negotiation cools down ideological intransigence and promotes pragmatic solutions. Cooperation in committees, government and parliament leads to mutual adjustments where learning processes occur over the substantive issues of legislation. Reaching a satisfactory compromise may take more time than a majority decision, but once the agreement becomes law most actors are prepared to accept it. This increases the chances of new laws and programmes being implemented (Abromeit and Pommerehne 1992; Armingeon 2003; Abromeit 1993; Poitry 1989) and makes for sustainable policy decisions (Hirschi et al. 2002).

Yet, criticism of consensus democracy is as old as its praise. Political scientists have noted that consociationalism renders a strong opposition impossible. Elections do not provide an opportunity for government and opposition to alter places as they do in pure majority democracies. Therefore, the Swiss system lacks the larger innovatory and social learning processes that are brought about by complete changes of power—it is not the country of revolutions but evolutions.

Two scholars proposed radical modifications to stimulate innovation. The political scientist Raimund Germann (1975) proposed scenarios for a comprehensive institutional transformation into a majoritarian parliamentary system. He focused on the problem of incrementalism in domestic politics and later European integration. In his view, the Swiss had to adapt to the much faster pace of decision-making in the EU (Germann 1990). The economist Silvio Borner (Borner et al. 1990, 153ff. and 169ff.; Borner and Rentsch 1997), in turn, focused on the negative economic impact of negotiation practices. In this view, the strong position of interest groups in the legislative process led Swiss enterprises towards seeking state rents instead of taking their chance on the market. Industries, getting short-term benefits from protectionism, would in the long run lose their capacity to innovate and compete on the international markets.

The proposition of both Germann and Borner was clear: more competition is necessary for Swiss politics and for the Swiss economy as well. Their message was appreciated neither by politicians nor the public. The reason was simple: both were honest enough to name the price of more political competition. Installing a bi-polar competitive system would require not only less direct democracy but also more centralisation and less bicameralism. Direct democracy and federalism, however, are sacrosanct in the eyes of both citizens and politicians. So, the Swiss stay with their consensus democracy.

## Consensus Democracy Under Stress

### The ‘Konkordanz’ Crisis of 2008

**Fig. 5.2:**
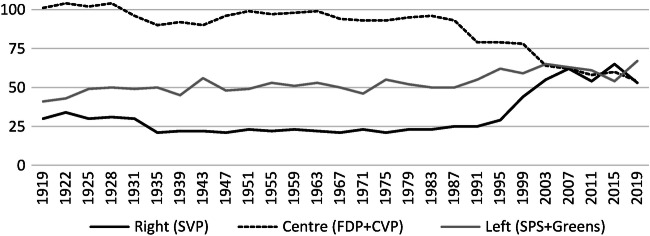
Seats in the National Council of the five main political parties, 1919–2019. (Source: own figure based on data from *Bundesamt für Statistik* [BFS; 2019])

The last three decades have brought increasing volatility in elections. Between 1991 and 2007, the Swiss People’s Party (*Schweizerische Volkspartei* [SVP]) more than doubled its share of the electorate, became the biggest political party nationwide and was given a second seat in the Federal Council in 2003. Their success came at the cost of the political centre, Radicals (*Freisinnig Demokratische Partei* [FDP]) and Christian-Democrats (*Christlichdemokratische Volkspartei* [CVP]), while the left with Social-Democrats (*Sozialdemokratische Partei der Schweiz* [SPS]) and Greens gained initially, then stagnated for a long time and eventually gained again at the 2019 elections (Fig. 5.2).

Many of the smaller parties did not survive, disappeared from the political arena or merged with others, such as the Liberals with the Radicals (FDP). Higher electoral volatility is nothing extraordinary as such, but it was accompanied by increasing political polarisation . The SVP, in taking over the old xenophobe parties, moved to the right and in many issues attacked not only the left but also the centre. This considerably changed the Swiss party system. The bourgeois alliance is partly broken, leading to a tri-polar system of political forces: a populist right, a green-socialist left and a fragmented centre.

The rise of the SVP began in 1992 when its de facto leader, Christoph Blocher, successfully mobilised against the European Economic Area (EEA) Treaty. It became the party of Eurosceptics and later systematically opposed the *Konkordanz* also on issues of immigration, social policy, and institutional reform. Originally confined to an electorate in the Protestant German-speaking cantons, the SVP went on to win new voters all over Switzerland and from all social strata. The party is much more professionalised in its organisation and has many resources for costly campaigns. It fosters its nationalist-conservative profile in an aggressive, often populist style (Mazzoleni 2003), thus becoming the political agenda setter in the media.

This began to amount to much more than ‘just’ issue-specific opposition. The other parties accordingly accused the SVP of betraying the spirit and workings of the *Konkordanz* . This was a blame levied particularly against Blocher. Elected in 2003 as the second representative of his party to the Federal Council (see above, Sect. 5.1.1), Blocher continued to act as an informal leader of the SVP. In late 2007, a coalition of Social-Democrats, Christian-Democrats and Greens successfully plotted his replacement by a more moderate SVP member. The reaction of the party was furious. It declared that it felt no longer represented in the Federal Council, excluded the two serving federal councillors elected as SVP members from its parliamentary group and declared ‘fundamental opposition’ to the government.

This incident developed into a real crisis of the *Konkordanz* , even if only for a rather short period.^4^ The SVP’s ‘fundamental opposition’ itself lasted only a year (Church and Vatter 2009). Its leaders realised that a single party alone could not break up the system of power-sharing: the institutional constraint for cooperation among the rest of the governmental parties was stronger. Thus, when one of the two (former) SVP ministers resigned in late 2008, the party was quick to claim his seat and even re-proposed Blocher, to whom most other parties still strongly objected. The result was a compromise in that not Blocher but the former leader of the party was elected—even if only just, with 122 votes against 121 for a more moderate SVP member (APS 2008).

The final step of the governmental reintegration of the largest political party occurred in late 2015, when the other former SVP member resigned. This time, Blocher signalled no interest at all and the party put forth three official candidates, one from each linguistic region. Eventually, the French-speaking Guy Parmelin was chosen, thus becoming the first SVP Federal Councillor speaking that language (APS 2015). Thus was restored the (adjusted) magic formula of 2003: two SVP, two SPS, two FDP and one CVP member. All government elections since then (in 2018 and 2019) have confirmed this distribution of seats.

### Power-Sharing in a Polarised Parliament

Growing political polarisation raises the question whether parliamentary compromise is still possible. Indeed, until the end of the 1980s, a relatively stable block of the three bourgeois parties had few difficulties to find a majority in cases of opposition from the left. With the partial disintegration of the bourgeois block, this seems no longer to be the case. The SVP acts as an issue-specific opposition almost as often as the Social-Democrats (Smartmonitor 2019), so the government coalition is exposed to opposition from both sides. No wonder that the media today often blame parliament for being incapable to reach a consensus.

**Fig. 5.3:**
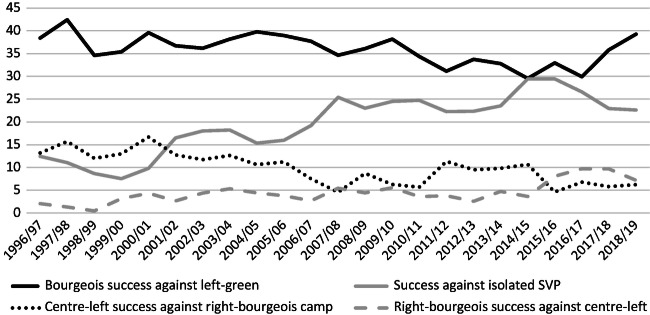
Most frequent winning coalitions in the National Council, 1996–2019 [%]. (Source: own figure based on data from Smartmonitor [2019]. Note: shown are the shares of different winning coalitions. Bourgeois parties = CVP, FDP and SVP; Centre-left = SPS [and Greens] and SVP; right-bourgeois = FDP and SVP)

An exhaustive analysis of about 8000 decisions of the National Council from 1995–2004, however, reveals a slightly different picture (Schwarz and Linder 2007). Blocked situations in which parliament cannot decide on a governmental proposition are, at least statistically, very rare, and parliamentary decisions are still characterised by manifold winning coalitions that vary from issue to issue. Particularly Christian-Democrats sometimes vote with the Social-Democrats and Greens, which means that the National Council is practising the game of power-sharing in a more open way than in the 1980s. In the long run, issue specific coalitions also change, an indication that political trends are more important than stark ideologies. Finally, the study shows that the political centre—the Christian-Democrats and the Radicals—is the most important policy shaping actor in the parliamentary arena. Their coalition is most successful in forging winning coalitions (Fig. 5.3). The centre also benefits from situations found in many controversial issues in which propositions from the left and from the conservative right cancel each other out. These findings contrast with public opinion, which perceives the SVP as the strongest force and agenda setter. The SVP has electorally benefitted from polarisation but in some way pays for its strategy of fierce and sometimes populist opposition with less influence in the parliamentary arena.

Foreign observers sometimes designate the SVP as the prototype of European populist parties. Indeed, the SVP was one of the first to be anti-elitist, and to denounce its political antagonists with a blunt rhetoric, claiming to be the only legitimate voice of the people. Constant fundamental opposition and sometimes radical proposals against European integration and immigration were key to its political success. The SVP became the favourite political party of all segments of society which were the real or imagined losers of modernisation or threatened by the process of globalisation. All of these elements resemble the populism of right-wing parties in other European countries.

Yet there are also substantial differences. The SVP has been part of the Swiss government for several decades, remaining bound and integrated into the ‘grand coalition’ with the other main parties. Also, the party does not have to call for *more* direct democracy to better reflect the will of the people. Instead, it used the instruments of direct democracy and in fact made the experience that it does not always represent ‘the will of the people’. Other parties complain about the populism and radical proposals of the SVP, but no one would try to exclude them from the pluralist discourse as we can observe in other countries. Instead, the other parties have learned to live with a ‘tamed’ form of populism, which is not only the privilege of the SVP. In that sense, the SVP is both emblematic and antinomic of populist parties elsewhere.

### The Pressure of Globalisation

Globalisation opens national economies, reduces economic protectionism, and stimulates market competition, liberalisation and privatisation. Politically, international authorities and supranational organisations become important regulators. The sharp distinction between domestic and foreign policy fades away, the national state loses autonomy and sees its own sphere of influence vanishing. Switzerland is exposed to all these general effects. In Chap. 10.1007/978-3-030-63266-3_3, we presented the particular situation of Switzerland in the process of Europeanisation. Good relations with the EU are paramount for the Swiss economy. In not being a member of the EU, the Swiss government tries to develop them by the way of ‘bilateral treaties’. It pays a high price for the bilateral way. Equal treatment of Switzerland with regard to its member states is a legitimate interest of the EU. This means that Brussels influences regulation much beyond the bilateral treaties, without the Swiss having any influence on the content and development of the EU’s *acquis communautaire*. Therefore, Switzerland today is highly integrated into the European market and has little chance not to do so. Globalisation in Switzerland, to a high degree, means Europeanisation. This has also changed the political structures and processes of power-sharing (Fischer 2007, 2014):

- The dynamics of EU economic integration put Swiss politics under permanent pressure. This may be one of the reasons why power-sharing, despite polarisation, is working.
- The agenda of Brussels is conceived in Swiss politics as an imperative for liberalisation, privatisation and economic reform. Europeanisation has changed the balance of powers. Export industries and parts of the consumer interests use ‘Brussels’ as their ally and have become stronger. In contrast, unions, farmers, artisan industries and other actors of the domestic market have lost a considerable part of their influence.
- Internationalised regulation in Switzerland exceeds domestic law in volume and growth. It is the government and its diplomacy which control the agenda in international relations and who are the actors of treaty making. Parliament is involved in early consultations but in cases of a treaty can only reject or accept the government’s proposition. Thus, it loses influence in many issues.

These developments have led to kind of a two-pace regime. Decision-making in globalised affairs has become different to conventional patterns of power-sharing. In the ‘globalised regime’, the executive is much more in the centre of the process. Some of the classical veto positions are weakened: pre-parliamentary consultation is more selective, vested interests of domestic policies have less bargaining power and the policy-shaping role of parliament is reduced. Federalism, the strongest veto position besides the referendum, can be overruled, as was illustrated in Sect. 3.5.3 of Chap. 10.1007/978-3-030-63266-3_3. In contrast to the incremental process in domestic issues, policy-shaping and -making in Europeanised affairs are developing a different pattern (Mach et al. 2003): innovation passes more quickly and makes bigger steps.

However, the shortcuts of this process, which bypass or reduce the veto power of many actors, have their price. Europeanisation and globalisation (re)produce many salient issues fuelling polarisation among the political elites and sometimes also between them and ‘ordinary’ people. Moreover, also the cleavages between urban and rural areas as well as between different social strata have become stronger, both in the perception of citizens as well as during certain popular votations (Linder et al. 2008; Seitz 2014; Swissvotes 2019).

## Conclusions

### Swiss Democracy: An Exceptional System

Thus far, we have discussed three main features of Swiss democracy: federalism, direct democracy and power-sharing. At first sight, these three are anything but Swiss particularities: worldwide, we count about 30 federal systems. Direct democracy is practised also in the individual states of the US, and power-sharing can be found in the Netherlands and Belgium as well. Moreover, direct democracy can combine with majoritarian democracy , and consensus democracy with a representative system (Table 5.2).

**Table 5.2:** Different types of democracy: some examples

Type of democracy	*Representative*	*Semi-direct*
*Majoritarian*	The UK New Zealand	States of the US
*Consensus*	The Netherlands Belgium	Switzerland

It is the *combination* of power-sharing and direct democracy that puts the Swiss system at odds with much political theory and mainstream political thought. In contrast to other countries like The Netherlands, for instance, Swiss consensus democracy is *not* the result of negotiations among the political parties after elections but a permanent institutional constraint due to the referendum. In the US, direct democracy is not practised at the national level as in Switzerland, nor has it led to power-sharing in its individual States. And while elections to parliament are the decisive element in the competition between government and opposition in the UK and New Zealand, they have no such effect in Switzerland. Thus, the same institutional elements may function differently in different contexts, which is why it is important to look at them as a whole.

Let us compare the most different countries of Table 5.2: the UK and Switzerland (Table 5.3). In the former, which provides an almost ideal example of a majoritarian and representative system, political power is concentrated in the hands of the political party that wins a majority.^5^ Intense election campaigns are linked to fierce interparty competition, and the winner ‘takes it all’. The electoral system is designed to bring about strong and stable parliamentary majorities, formed of usually just one party. The elected government sets out to implement the programme it had laid before the electorate, but if it fails to carry its programme through the opposition has a chance to hold it accountable and form a new government in turn. Strong innovation is possible, also against the opposition. The influence of voters can be described as programmatic, since it is they who choose among the programmes of the major parties and thus define the political agenda for four to five years. Sometimes party manifestos propose major policy changes, so elections provide popular legitimacy, policy innovation and political change at the same time.

**Table 5.3:** A system comparison between Great Britain and Switzerland

Great Britain: *Representative, majoritarian democracy*	Switzerland: *Semi-direct, consensus democracy*
Strong competition between parties Winner takes it all	Weak party competition Proportional representation
Salient elections lead to periodical alternation of power	Low salience of elections; power-sharing amongst political parties prevents alternation of power
Enactment of the political programme of the government, backed by a parliamentary single-party majority	Integration of cultural minorities and conflicting group interests; changing coalitions for different issues
Big innovation possible	Incremental innovation only
Political legitimacy through changes in power or re-election of a government satisfying voters’ expectations	Institutional legitimacy through different forms of participation: the most important decisions are taken by the people, important ones by parliament and the rest by the government
Underlying idea: politics for the people	Underlying idea: politics through the people
Participation as a form of general and programmatic influence: voters elect a government and its programme for the entire legislative period	Direct participation as ‘single-issue’ influence: people vote on specific questions. No strategic government policy, no influence of voters on a specific government programme

In a semi-direct consensus democracy, on the other hand, party competition is low because elections cannot lead to a change of roles between government and opposition. The system places its trust in the final control by the people over all important issues. Legitimacy comes from the most important decisions being taken by the people directly. Proportionality in elections and mutual adjustment in legislative decision-making favour the idea of ‘no single winner takes everything, everybody wins something’. In direct democracy, voting is on a single issue at a time, and each case produces different winning coalitions, which are barely foreseeable by the political elites. A popular vote, even when settling a fundamental issue, involves just one clear decision independent from the others. The Swiss government, free from the fear of not being re-elected, will not spend much time on programmatic strategy. The narrow limits of manoeuvre imposed by an all-party government and the permanent risk of a referendum defeat drastically curtail any effort to design comprehensive programmes and, at least in domestic policies, allow for incremental progress only.

Both countries, in their particularities, are unique. But while the UK and its Westminster system have become a mainstream model for democracy all over the world, the Swiss polity with its combination of direct democracy and power-sharing has remained exceptional. Swiss democracy is at odds with the prevailing idea of democracy as *just* a competition among elites.

### Who Has More Influence: The British or the Swiss Voter?—The Trade-off Between Elections and Direct Participation

The comparison between the UK and Switzerland has revealed fundamental differences in the way the idea of democracy as ‘rule by the people’ is realised. These are not only differences of ‘systems’ but also differences of how citizens can influence politics. A British voter chooses—by means of her ballot for a single candidate—which political party, its leader and its programme should be confirmed or voted out of office. The electoral choice of every British voter is of utmost importance. Victory or defeat of a party in parliamentary elections determines the political future of the country, and even London stock markets react by going up or down. Between two elections, however, the British voter has little to say, and the ruling government is not too much impressed by bad records of popularity in surveys. The prime minister, as long as her majority in parliament is not put at risk by a vote of no confidence, has little to fear from the polls. That should provide ample time to implement what she promised before the elections.

In contrast, the Swiss voter knows that after the elections there will most probably be the same four-party government as before. Electoral swings may lead to some changes in the relative influence of parties in parliament and even minor adjustments in the composition of government. But looking back at the last 60 years, the voter can rest assured that an all-party government, composed proportionally to the relative strength of the biggest parties, will be in power. The Swiss stock market is not impressed either. Between two elections, however, the Swiss voter additionally exercises her rights of direct participation: saying ‘yes’ or ‘no’, she has the last word on many important decisions prepared by parliament. Obviously, British citizens have maximal electoral influence, but no say through direct-democratic choice. For Swiss citizens the reverse is true. This raises two questions.

**Fig. 5.4:**
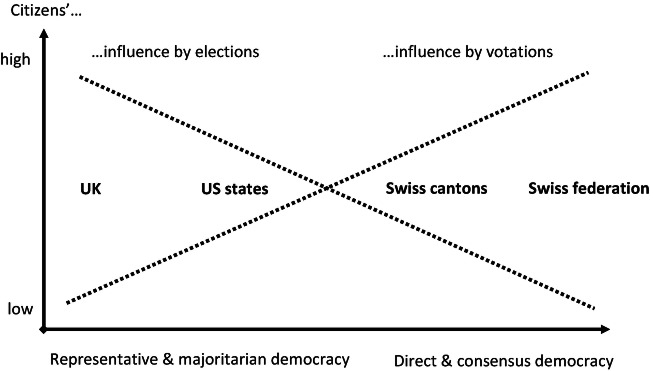
Citizens’ influence in majoritarian parliamentary and semi-direct consensus democracies: a theoretical model

The first is, could we have it both ways, that is: having a maximum of influence through elections *as well as* by direct participation? The answer is no. While the idea of more democratic influence by both elections and direct democracy is tempting, it simply cannot be realised within the same political system. In Switzerland, an institutional change to more electoral competition is possible only by reducing the import of direct democracy, notably the referendum which requires parliamentary and governmental power-sharing. Similarly, regular referenda in Great Britain would destroy the basic idea of its political system, namely to concentrate power in the hands of a strong government which, based on its parliamentary majority, can realise its programme also against the will of the opposition. There is a trade-off between influence by elections and direct participation: the more a political system realises high voter influence by elections, the less it can grant influence by direct participation, and vice versa (Linder 1991, 49). Figure 5.4 visualises this trade-off and locates some exemplary polities.

At the either end of the spectrum ranging from representative and majoritarian to direct and consensus democracy, we locate the UK and Switzerland, each of which maximises influence through one of the two forms of participation—elections or votations—while offering the least influence through the other. Between these two there are some intermediate types. Swiss cantons differ in their extent of direct democracy. Yet all of them offer a higher degree of influence through elections than the federal level because also the executive branch—the cantonal governments—is elected by the people. The US states are situated more on the side of representative-majoritarian democracy. As in the UK, the competitive and majoritarian election of state legislatures and governors provide an opportunity for complete political change, yet in many US states we also find a frequent use of the initiative and referendum.

The hypothesis of an institutional trade-off between elections and direct democracy is in contrast to arguments of US political scientists Tolbert and Smith (2005), who argue that the political culture of direct democracy has a positive effect on electoral participation. However, Stadelmann-Steffen and Freitag (2009), in their exhaustive analyses of the Swiss cantons, confirm this trade-off: the more open a canton to direct democracy, the lower electoral participation.^6^

The trade-off means that voters cannot have the maximum of political influence by both elections and direct participation. This leads us to the second question: which combination of the two is ‘best’ in terms of maximum voter influence? This is not easily answered because we would have to know how citizens themselves evaluate these two forms of influence. The fact that social movements in many European countries seek some forms of direct democracy may be a sign that majoritarian parliamentary systems today need some complement to their purely electoral democracy. It is obvious, however, that regular referenda in Britain, for instance, would weaken not only the ability of government to achieve its programmes, but also depress the importance of elections, which would be a disenchantment for the British voter. Similarly, competitive elections in Switzerland could give more influence to the electorate, but the same electorate would not accept cut-backs in direct democracy. There is no panacea, and all we can do therefore is to look for ‘optimal’ voter influence, which depends on further particularities of a polity. In other words: finding combinations of single-issue direct participation and programmatic elections that in the eyes of the electorate best serve them to shape their own democracy.

### Consensus Democracy: Its Past and Its Future

Looking at the past, we can distinguish three different features of consensus democracy. The first is integration, which in the twentieth century had different meanings. In earliest times, power-sharing helped overcome the religious divide and prevented the linguistic minorities to be dominated by the German-speaking majority. Later, the social partnership and governmental inclusion of the political left helped reduce class conflicts. In the most crucial period before World War II, a high national consensus helped overcome threats to the country’s independence.

The second feature is political stability and efficiency. The perfection of power-sharing after World War II was undoubtedly beneficial. Switzerland passed smoothly through growing wealth to societal modernisation: the stability of its political system was an advantage for its economy in many respects. Whereas some West-European democracies went from liberalism to socialism and back, Swiss politics held its middle course. The policy of integration and prudent adaptation rather than risky innovation proved effective.

The third one is the development of a specific political culture. British scholar Clive Church, already mentioned as a life-long observer from the outside, provides a definition that goes well beyond the scope of conventional surveys (see Box 5.4). Many of these items are closely related to power-sharing. We may leave open the question whether this political culture was influenced by Swiss institutions or vice versa. The important point is that the functioning of power-sharing also depends on the cultural attitudes and political willingness of political elites and citizens alike.

#### Box 5.4 Swiss Political Culture, as Defined by a British Scholar

Cognitive Limited collegiate authority

Constitutionalist

Decentralist and federalist

Democratic

Neutral

Pluralist

Republican

Affective Desire for decisions to be made directly

Enthusiastic support for federal and other institutions

Multiple loyalties

Positive belief in compromise and cooperation

Strong sense of patriotism and independence

Tolerance of domestic differences

Willingness to accept adverse decisions

Judgemental Acceptance of the obligation to take part in politics

Agreement that nation depends on acts of will

Belief that the country is fragile

Cautious attitude to policy change

High levels of satisfaction with outcomes

Positive evaluation of Swiss democracy and neutrality

Trust in authorities

Source: Church (2004, 183)

It would be wrong, however, to overlook the shadows of consensus democracy which began to grow longer since the 1990s. Economic recession made political consensus more difficult. In such periods the lack of innovation and coherent government policies was particularly felt. Political power-sharing does not include foreign residents who represent 25% of the population, and efforts at social integration were insufficient. Moreover, new social movements—progressive as well as conservative (cf. Kriesi 1995)—indicated a loss of the Swiss system’s capacity to integrate all parts of society. The basic consensus among the political elite vanished, and the defeat of the government in the vote of 1992 on joining the European Economic Area left a divided nation.

Since the 1990s, pressure from the outside in the form of globalisation and Europeanisation has stimulated innovation. Power-sharing, despite growing polarisation and the crisis of 2008, is still working. The grand government coalition is sometimes defeated in referenda, but not more often than in earlier times. In parliament, growing antagonisms between the national-conservative right and the progressive-interventionist left are compensated by changing issue-specific coalitions in which the political centre plays an important role. The partial break-up of the bourgeois camp has made this possible. Under the conditions of a tripartite system of the right, the centre and the left, consensus democracy has the chance to work even better than in the 1980s, when the bourgeois majority made the left a permanent loser.

Informal rules play a prominent role for Swiss consensus democracy. The spirit of these rules is more important than their strict application. So in a short period of time, namely in 2003 and 2007, parliament twice broke with the unwritten rule that serving ministers are re-elected if they so wish. In both instances it meant saving one of the most important elements of Swiss democracy, namely a functioning and inclusive government. In 2003, parliament adjusted the magic formula to match the respective parties’ electoral strength. In 2007, it affirmed that Swiss government members are elected to practice collective leadership, not to implement their party’s electoral pledges. The electoral success of the Greens in 2019 did not lead to personal changes of the Federal Council—with the effect that at present, the principle of proportional representation is not fully respected.

Problems for the future remain. There are strong indications that decision-making on issues exposed to globalisation follows a different pace. Europeanisation, especially, leads to quicker and bigger innovations but bypasses many of the veto positions and interests relevant in domestic politics. This could lead to further polarisation between winners and losers of globalisation. Another problem is governmental reform. In a globalised world, the Federal Council and its administration have become key players, but the collegiate structure of the council—seven members with equal competencies—is still the same as in 1848.

Institutionally, consensus democracy has proven its worth in stormy weather. Surveys show that consensus democracy gets rising popularity and is even more appreciated by ordinary citizens than by the Swiss elites (MIS Trend 2008; Credit Suisse 2018). Here, however, we may identify the real weak spot of Swiss consensus democracy today. Polarisation, stimulated by the political parties of the right and of the left, leaves its traces in political culture. Pluralism, belief in compromise and cooperation, tolerance towards differences and willingness to accept adverse decisions are declining among parts of the political elite, and parts of the electorate as well.

With good reason, adherents of the Swiss *Konkordanz* worry about the loss of the ‘spirit of accommodation’. It could paralyse power-sharing in the long run. As a strategy towards majoritarian politics, however, the politics of confrontation would not be enough. A gradual transformation towards majoritarian politics seems feasible only given electoral change which sees a leading party, capable to formulate a convincing political programme but also to carry out the necessary institutional reforms. And the trade-off mentioned earlier will impose limits to such a transformation: in the near future, one should not expect the Swiss to be willing to abandon consensus democracy in favour of a majoritarian system with less direct democracy.

## Footnotes

1 Largely based on Aubert (1982, 1122ff.).

2 The parliamentary initiative is to be distinguished from the popular initiative, which emanates from outside parliament—although frequently used by political parties to mobilise their followers (Leemann 2015).

3 For a recent special issue devoted to consociationalism entirely, see Bogaards and Helms (2019).

4 As Widmer & Häusermann (2018) rightly observe, the willingness of political parties to compromise varies greatly from year to year. Years of low agreement, notably between 2003 and 2015, have been called ‘discordance’ (Widmer 2013, 234; Widmer 2015, 294). We doubt, however, if this concept is of analytical value because we cannot see significant correlations with the political events leading to a crisis of the consensus system. More importantly, ‘concordance’ is an institutional arrangement. None of its elements—with the exception of one or two partisan seats on the Federal Council—have ever been seriously put into question (cf. also Vatter 2016, 72; O’Leary 2019, 570).

5 We are aware that at the time of writing (October 2019), these statements need some qualification. Yet it is probably not by coincidence that the current chaos is due to three ‘un-British’ elements, namely direct democracy (the Brexit vote of summer 2016), lack of a parliamentary majority (the so-called hung parliaments returned in both 2010 and 2017) and the Fixed-Terms Parliament’s Act of 2011, which curbs the power of the prime minister to call an early election.

6 A comparison of all 26 cantons makes sense because although both government and parliament are popularly elected everywhere, there is a wide difference in the extent of direct democracy. For instance, Glarus and Appenzell Inner-Rhodes still practice the *Landsgemeinde*, an annual citizen assembly comparable to US town meetings. In turn, notably in the French-speaking cantons the representative element is much stronger (cf. Bühlmann et al. 2014).
